# Enhanced recruitment of genetically modified CX3CR1-positive human T cells into Fractalkine/CX3CL1 expressing tumors: importance of the chemokine gradient

**DOI:** 10.1186/s40425-016-0125-1

**Published:** 2016-04-19

**Authors:** Imran Siddiqui, Marco Erreni, Mandy van Brakel, Reno Debets, Paola Allavena

**Affiliations:** Department of Immunology and Inflammation, Humanitas Clinical and Research Center, 20089 Rozzano, Milan Italy; Laboratory of Tumor Immunology, Department Medical Oncology, Erasmus MC Cancer Institute, 3000 CA Rotterdam, The Netherlands; Ludwig Center for Cancer Research, Department of Oncology, University of Lausanne, 1066 Epalinges, Switzerland

**Keywords:** Adoptive T cell therapy, Chemokines, Chemokine gradient, CX3CR1, Colorectal cancer

## Abstract

**Background:**

Adoptive T-cell based immunotherapies constitute a promising approach to treat cancer, however, a major problem is to obtain effective and long-lasting anti-tumor responses. Lack of response may be due to insufficient trafficking of specific T cells to tumors. A key requirement for efficient migration of cytotoxic T cells is that they express chemokine receptors that match the chemokines produced by tumor or tumor-associated cells.

**Methods:**

In this study, we investigated whether the in vivo tumor trafficking of activated T cells could be enhanced by the expression of the chemokine receptor CX3CR1. Two human colorectal cancer cell lines were used to set up a xenograft tumor model in immunodeficient mice; the NCI-H630, constitutively expressing the chemokine ligand CX3CL1 (Fractalkine), and the RKO cell line, transduced to express CX3CL1.

**Results:**

Human primary T cells were transduced with the receptor CX3CR1-eGFP. Upon in vivo adoptive transfer of genetically modified CX3CR1-T cells in mice bearing NCI-H630 tumors, enhanced lymphocyte migration and tumor trafficking were observed, compared to mice receiving Mock-T cells, indicating improved homing ability towards ligand-expressing tumor cells. Furthermore, significant inhibition of tumor growth was found in mice receiving modified CX3CR1-T cells. In contrast, tumors formed by RKO cells transduced with the ligand (RKO-CX3CL1) were not affected, nor more infiltrated upon transfer of CX3CR1-T lymphocytes, likely because high levels of the chemokine were shed by tumor cells in the systemic circulation, thus nullifying the blood-tissue chemokine gradient.

**Conclusions:**

This study demonstrates that ectopic expression of CX3CR1 enhanced the homing of adoptively transferred T cells towards CX3CL1-producing tumors, resulting in increased T cell infiltration in tumor tissues and decreased tumor growth. Our results also establish that a correct chemokine gradient between the systemic circulation and the tumor is an essential requirement in adoptive T-cell based immunotherapy to efficiently recruit T cell effectors at the correct sites.

**Electronic supplementary material:**

The online version of this article (doi:10.1186/s40425-016-0125-1) contains supplementary material, which is available to authorized users.

## Background

Cancer immunotherapy involves the harnessing of the immune system to recognize and destroy tumor cells, and is an increasingly growing area of research to combat different types of cancer [1]. In the last decade, there has been a tremendous increase in optimizing this approach in order to make it a clinically feasible treatment. Adoptive T cell therapy (ACT), one of the major modalities within cancer immunotherapy, is a potentially powerful approach to cancer treatment based on the infusion of tumor specific T cells [2–4].

ACT has multiple advantages over other forms of cancer immunotherapy, and relies on the transfer of ex vivo activated and sometimes gene-engineered T cells, which encounter and act against tumor antigens in patients, mediating tumor regression in variety of cancer such as melanoma [5], cervical cancer [6], lymphoma [7], leukemia [8], bile duct cancer and neuroblastoma [9]. There are many ways to enhance and optimize the ACT approach, such as isolating and re-educating tumor infiltrated lymphocytes in IL-2-containing medium, or genetically modifying T cells to express antigen-specific T cell receptors (TCRs). T cells have been engineered to express on their surface, chimeric antigen receptors (CARs) as well as CARs including co-stimulatory molecules. Such approaches have been effectively employed as a robust treatment for cancer with greater number of patients responding to therapy [10, 11].

However, one of the major limitations of ACT is the failure to induce long-lasting responses, possibly because an insufficient number of tumor-reactive T cells reach the tumor [12]. It has been suggested that appropriate trafficking of cytotoxic T lymphocytes to the tumor site is a critical step to obtain anti-tumor responses, as the presence of increased tumor-infiltrating lymphocytes (TIL) has been reported to correlate with better clinical outcomes [13, 14]. Of note, ACT appears successful against hematological cancers, a result that underlies the importance of proper T cell infiltration. T cells are recruited from the circulation in a series of distinct processes, involving adhesion to endothelium, chemotaxis and extravasation [15]. The critical steps of tissue penetration are dependent on different factors such as expression of adhesion molecules on endothelium and lymphocytes [16] and of chemo-attractants produced by tumor tissues [17]. Considering the major role of chemokines and their receptors in regulating and directing the tissue trafficking of T lymphocytes, we explored the feasibility of transducing a chemokine receptor in human T lymphocytes and investigated their tumor homing ability upon adoptive transfer in tumor-bearing mice.

Some studies have shown significantly higher infiltration of T cells with better migration potential when modified to express relevant chemokine receptors on their surface [18–26].

In this study, we focused on the chemokine Fractalkine/CX3CL1, the unique member of the CX3-chemokine subfamily, which exists as a membrane-bound molecule, in addition to the cleaved soluble form. Fractalkine is expressed in several types of cancer including pancreatic [27], breast [28], gastric and colon cancer [29–32]. Through its specific receptor CX3CR1, it strongly induces chemotaxis and migration of NK cells, Th1 lymphocytes and macrophages [33, 34].

Here we show that the CX3CR1 receptor can be efficiently transduced in human T cells; we have set up an adoptive transfer model in NOD-scid IL-2Rgamma^−/−^ (NSG) mice bearing a human colorectal cancer cell line constitutively expressing the ligand CX3CL1. Specific enhanced recruitment of CX3CR1-T cells towards CX3CL1-expressing tumor cells was observed, which lead to reduced tumor growth. Thus, the re-direction of T lymphocytes by chemokine receptors is feasible and valuable, and could be exploited to maximize the currently available therapeutic resources.

## Results

### Expression of the chemokine Fractalkine/CX3CL1 in human colo-rectal cancer specimens and cell lines

**Fig. 1:**
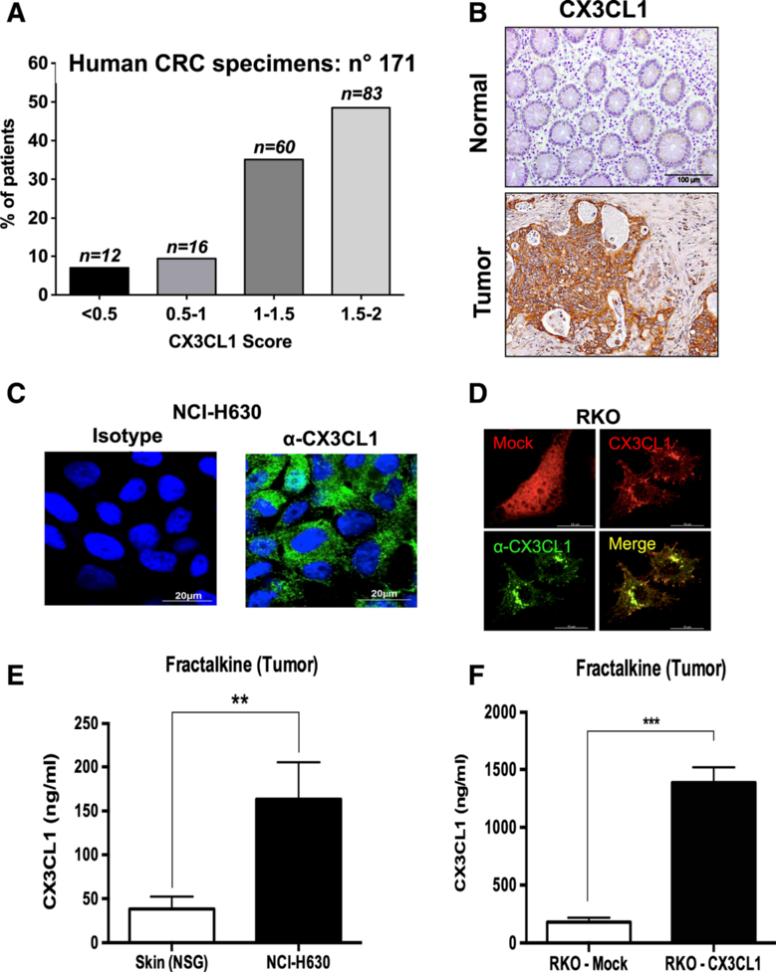
Expression of Fractalkine/CX3CL1 in human colon cancer tissues and cell lines. **a** Immunostaining score of Fractalkine/CX3CL1 in 171 patients with colorectal cancer; numbers on top of bars define exact number of patients with a given score; **b** Immunostaining of Fractalkine/CX3CL1 in human normal colonic mucosa (*top*) and in a representative human colon cancer tissue (*bottom*); **c** Immunofluorescence by confocal microscopy of Fractalkine/CX3CL1 protein expression in NCI-H630 cells and **d** in RKO cells upon transduction of the Fractalkine cDNA (CX3CL1-cherry) or mock-transduced (mock-cherry); **e** Quantification by ELISA of Fractalkine/CX3CL1 levels in the lysate of NCI-H630 tumors grown in NSG mice, normal skin tissue was used as reference control; **f** ELISA of Fractalkine/CX3CL1 levels in tumor lysates obtained from RKO tumors (RKO-MOCK and RKO-CX3CL1) grown in NSG mice

The chemokine Fractalkine/CX3CL1 is expressed in several types of cancers and strongly induces chemotaxis and migration of Th1 lymphocytes, NK cells and macrophages [30, 33, 34]. We and others reported that this chemokine is highly expressed in tumor cells of human colorectal cancer (CRC) [30–32, 35, 36]. Figure 1a shows that from a series of 171 CRC samples studied by immunohistochemistry, 83.6 % were positive or strongly positive for CX3CL1 and only 16.4 % had faint or negative immunostaining. Of note, the normal non-involved colonic mucosa was negative or weakly positive, as shown in Fig. 1b, which also depicts a representative picture of a CX3CL1-positive tumor sample.

Having determined that the majority of human CRC samples express the ligand Fractalkine, we next selected the most appropriate colorectal cancer cell lines to set up a tumor model to perform in vivo adoptive transfer experiments. It is known that in vitro established cancer cell lines frequently lose the ability to produce specific chemokines, as we reported for Fractalkine in pancreatic cancer [37]. We examined the expression of CX3CL1 in several colon cancer cell lines (Additional file 1: Figure S1A and B). In RKO and HCT116 cells, the mRNA levels were low and not inducible by IFN-γ and TNF-α, while were constitutively detectable in NCI-H630 cells and higher after stimulation (Additional file 1: Figure S1A). We decided to use the native NCI-H630 cell line and to transduce RKO cells with the cDNA of the CX3CL1 gene. After transduction with the Fractalkine gene fused with a Cherry vector, RKO cells did express high levels of mRNA levels, which were further induced by IFN-γ and TNF-α stimulation (Additional file 1: Figure S1B). Protein expression in NCI-H630 and RKO cells was confirmed by immunofluorescence (Fig. 1c-d).

To set up the in vivo mouse model, NCI-H630, RKO-Mock and RKO-CX3CL1 cells were inoculated s.c. in immunodeficient NSG mice. Growth of RKO-CX3CL1 cells was identical to that of RKO-Mock cells, indicating that the ectopic expression of CX3CL1 is not altering tumor cell proliferation, as shown in Additional file 1: Figure S1C. After three weeks, tumors were harvested to measure Fractalkine production in tumor tissues. The ELISA assay demonstrated significantly higher amounts of Fractalkine in the tumor lysates of NCI-H630 tumors, as compared to skin lysates, used as a reference of normal tissue (Fig. 1e); as expected, tumors formed by RKO-CX3CL1 produced much higher amounts of Fractalkine (Fig. 1f).

### Transduction of the receptor CX3CR1 in human T lymphocytes enhances their migration towards its ligand Fractalkine/CX3CL1

To optimize T cell trafficking into tumors producing the chemokine Fractalkine, we transduced human T cells to be adoptively transferred, with the chemokine receptor CX3CR1.

**Fig. 2:**
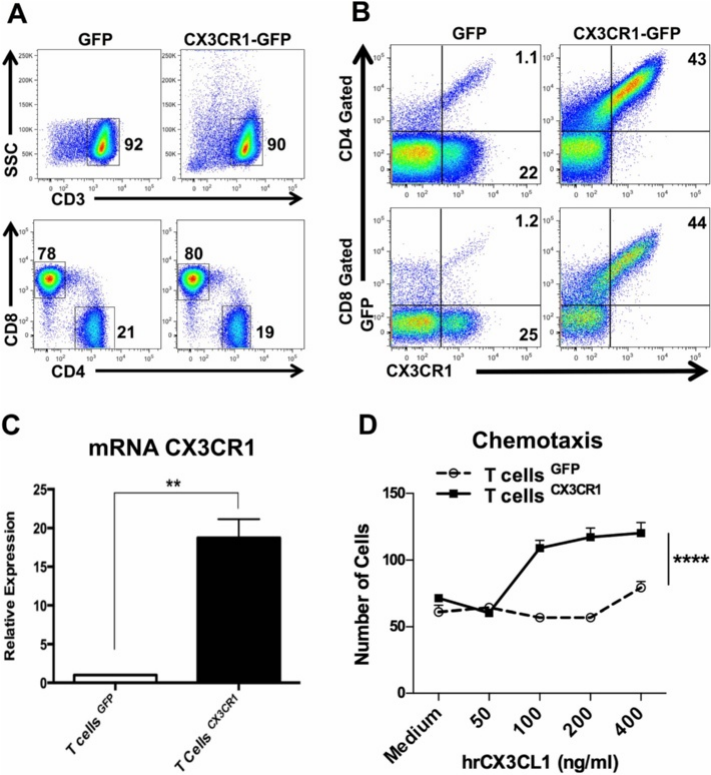
Transduction of the CX3CR1 receptor in human T cells and functional validation. **a** Population of CD3+ and of CD4+/CD8+ T cells post retroviral transduction of eGFP/CX3CR1 or -eGFP gene after 10 days of in vitro culture with hIL-2 medium. **b** Representative flow cytometry plot showing the transduction efficiency in eGFP or CX3CR1-eGFP in human T cells. Cells were gated on live cells followed by subsequent gating on CD3, CD4 and CD8 positive T cells. **c** mRNA expression of CX3CR1 in transduced CD3/IL-2 activated human T cells (Bars, Triplicates +/−SEM *P < 0.05, **P < 0.01 for difference between eGFP and CX3CR1-eGFP-T cells (Student’s *t* test). **d** Transwell migration assay of eGFP-T cells or CX3CR1-eGFP T cells in response to different concentrations of rhCX3CL1, ****P < 0.0001, Two-way ANOVA

The cDNA encoding CX3CR1-eGFP was cloned in the retroviral plasmid pMP71 and CD3-activated human T lymphocytes were transduced and further expanded in IL-2-containing medium. Three days post viral transduction, cells were >80 % CD3+ with a mixture of CD4 and CD8 lymphocytes (Additional file 2: Figure S2A); after 10 days, >90 % of cells were CD3+, indicating that the vast majority of IL-2-expanded cells were T lymphocytes, and 80 % were CD8+ (Fig. 2a).

Expression of CX3CR1 and its cell surface localization were assessed by flow cytometry using anti-human CX3CR1 antibodies. The transduction efficiency in the CD3+ T cell population, expressing both eGFP and CX3CR1, was confirmed in the CD4+ subset (43 %) as well as in CD8+ cells (44 %) (Fig. 2b). Expression of CX3CR1 was also determined by quantitative real-time PCR and found to be significantly higher in CX3CR1-eGFP-transduced cells compared to control T lymphocytes transduced with eGFP vector alone (Fig. 2c). We then performed a functional analysis of CX3CR1-expressing T lymphocytes. The classical migration assay was set up using recombinant human Fractalkine. The results showed that CX3CR1-T lymphocytes had significantly high and dose-dependent migration ability toward Fractalkine, whereas T cells expressing only eGFP did not show improved migration (Fig. 2d), confirming that the transduced receptor was fully functional.

### In vivo adoptive transfer of CX3CR1-transduced human T lymphocytes results in enhanced migration towards Fractalkine-expressing tumors

To address the homing and migration ability in vivo of CX3CR1-T lymphocytes towards tumors expressing the ligand, we performed adoptive T cell transfer experiments.

**Fig. 3:**
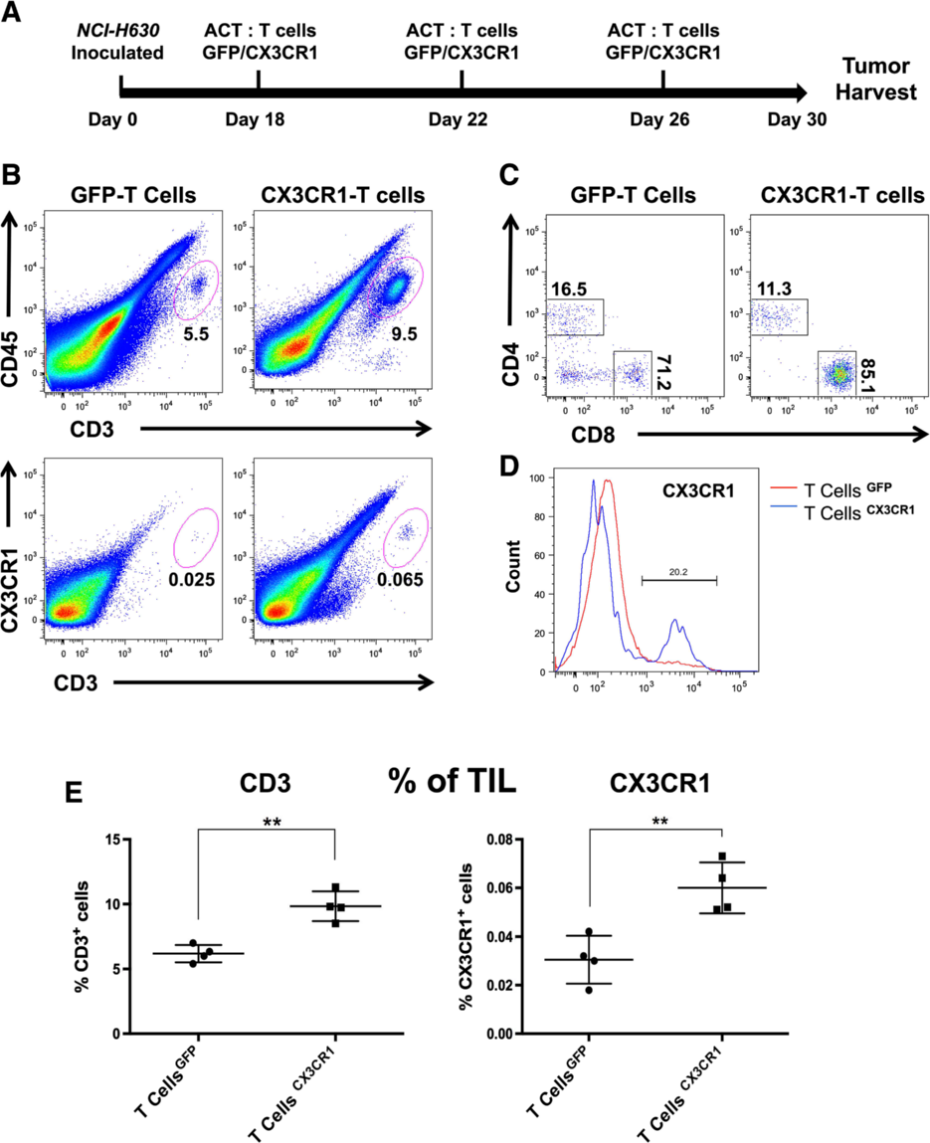
Adoptive transfer of CX3CR1-positive T cells to mice bearing NCI-H630 tumors. **a** Schematic representation of adoptive transfer of eGFP or CX3CR1-eGFP expressing T cells in NSG mice implanted with NCI-H630 tumors. **b** Representative flow cytometry plot of explanted and disaggregated tumors for the analysis of infiltrating human T lymphocytes (CD45 + CD3+/CX3CR1+) after adoptive transfer. Cells were gated on live cells, the plots show the population of CD3/CD45 positive cells and CD45/CX3CR1 positive cells within the tumors. **c** Proportion of CD8+ and CD4+ subpopulation within CD3 positive TILs. **d** Proportion of CX3CR1+ T cells in gated CD3+ tumor-infiltrating T cells (blue line: mice receiving CX3CR1-expressing T cells, red line: mice receiving eGFP-T cells CD3). **e** Summary of percentage of total CD3+ (*left*) and CX3CR1+ (*right*) T cells in explanted tumors after adoptive transfer, obtained from different mice (each dot represents a single mouse). +/−SEM **P < 0.01 for difference between control (eGFP) and test (CX3CR1) group (Student’s *t* test)

Mice were subcutaneously inoculated with NCI-H630 tumor cells and checked for tumor development; after 18 days, eGFP or CX3CR1-eGFP lymphocytes (3 × 10^6^) were transferred through tail vein injection. Mice received transduced T lymphocytes every three-four days (Fig. 3a). After three consecutive adoptive transfers, mice were sacrificed and tumors harvested, digested enzymatically and tumor-infiltrating lymphocytes (TIL) were analyzed by quantitative real-time PCR and by flow cytometry. The representative cytometry analysis, illustrated in Fig. 3b, shows high CD3+ and CX3CR1+ cells (within CD45+ gated population) of mice receiving CX3CR1- transduced lymphocytes, compared to mice receiving eGFP-lymphocytes.

**Fig. 4:**
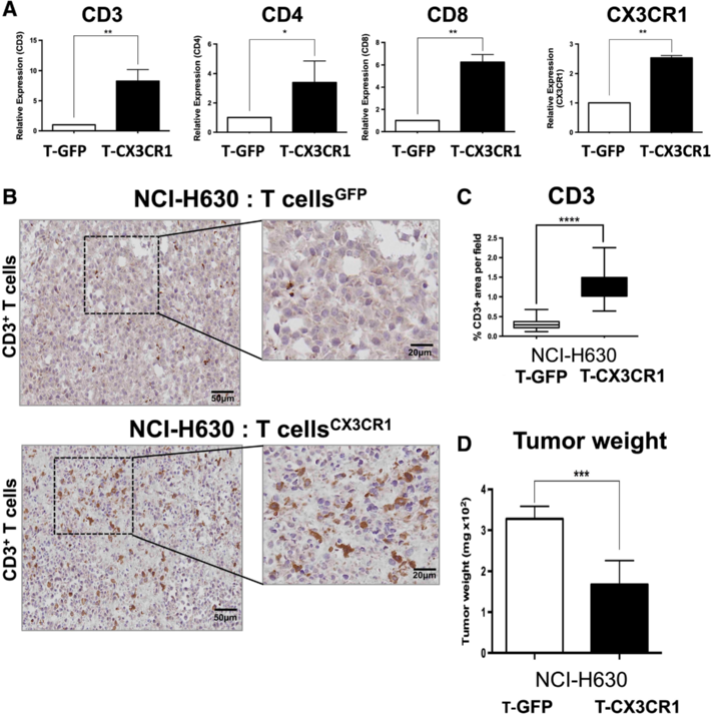
Analysis on tumor infiltrating human T cells after adoptive transfer to mice bearing NCI-H630 tumors. **a** mRNA expression of CD3, CD4, CD8 and CX3CR1 (human specific primers) from tumors of mice receiving eGFP-T cells (white bars) or CX3CR1-eGFP T cells (black bars), triplicates +/−SEM. **b** Immunohistochemical analysis of CD3 expression in paraffin embedded tumors after adoptive T cell transfer; **c** CD3 stain positive area quantified using image pro analysis software. **d** Weight of tumors after adoptive transfer of eGFP/CX3CR1-eGFP lymphocytes in mice (6–7 mice per group). *P < 0.05, **P < 0.01, ***P < 0.005, ****P < 0.001 for difference between eGFP and CX3CR1-eGFP expressing T lymphocytes (Student’s *t* test)

The proportion of CD8+ and CD4+ subpopulation within CD3 positive TILs demonstrated that up to 85 % of cells expressed CD8 (Fig. 3c); furthermore, a greater proportion of CX3CR1+ T cells were present within the CD3-gated population (Fig. 3d). The FACS analysis obtained from four distinct mice showed significantly higher infiltration of CD3+ and CX3CR1+ lymphocytes in tumors of each mouse receiving CX3CR1-T lymphocytes, confirming their preferential tumor homing ability (Fig. 3e). The Real-time quantitative PCR also demonstrated significantly higher mRNA levels of T cell markers (CD3, CD4, CD8 and CX3CR1) in tumors of mice injected with CX3CR1-T cells (Fig. 4a). The presence of TIL was also investigated by immuno-histochemistry in tumor sections. We observed higher number of CD3 positive T cells in tumors of mice adoptively transferred with CX3CR1-T cells compared to mice receiving eGFP- T cells (Fig. 4b and c). Finally, the harvested NCI-H630 tumors were measured and we found significant reduction in tumor weight in mice injected with CX3CR1-T cells, indicating effective anti-tumor activity of receptor positive T lymphocytes (Fig. 4d).

**Fig. 5:**
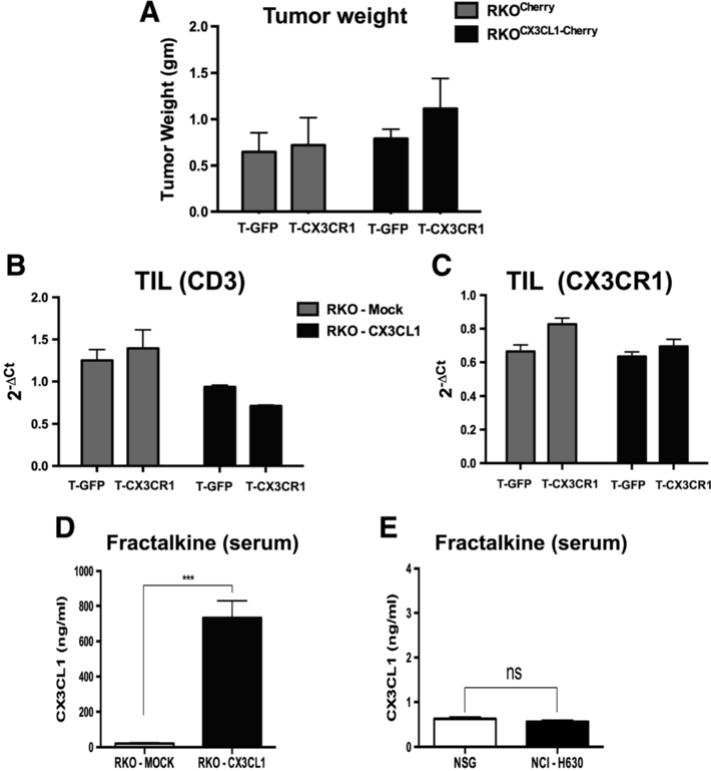
Adoptive transfer of CX3CR1-positive T cells to mice bearing RKO tumors over-expressing Fractalkine/CX3CL1. **a** Weight of RKO-Mock or RKO-CX3CL1 tumors after adoptive transfer of GFP-T cells or CX3CR1-T cells. (**b**, **c**) mRNA expression of CD3 and CX3CR1 (human specific primers) from RKO-Mock or RKO-CX3CL1 tumors receiving GFP-T cells or CX3CR1-T cells. **d** Quantification of Fractalkine in the blood serum of mice bearing RKO-Mock or RKO-CX3CL1 tumors. **e** Quantification of Fractalkine in the serum of mice bearing NCI-H630 tumors or in non-tumor-bearing mice

We repeated the same type of experiment in mice bearing tumors formed by RKO-CX3CL1 or RKO-Mock cells. Surprisingly, after transfer of CX3CR1-T lymphocytes we did not observe any reduction in tumor weight (Fig. 5a), nor were the tumors more infiltrated by T cells, as evident from CD3 and CX3CR1 mRNA expression in isolated tumor infiltrating cells (Fig. 5b, c). We suspected that the chemokine Fractalkine could be possibly shed in the circulation by RKO-CX3CL1 cells, thus abrogating the chemokine gradient between tumor tissues and the systemic circulation. Serum levels of Fractalkine in mice bearing RKO-CX3CL1 tumors were in fact very high (700 ng/ml) (Fig. 5d) while less than 1 ng/ml was detected in the sera of mice bearing NCI-H630 tumors (Fig. 5e). Furthermore, the lymphocyte analysis from single cell suspension of lung tissues, after adoptive transfer regimen, showed significantly more CD3 lymphocytes entrapped in the lungs of mice bearing RKO-CX3CL1 tumors compared to RKO-Mock tumors : 70 % CD3+ vs 50 %, (Additional file 3: Figure S3A). Of note, no significant difference was observed in the lung infiltrate of NCI-H630 tumors (Additional file 3: Figure S3B).

This finding emphasizes the importance of an appropriate chemokine gradient to efficiently recruit lymphocytes towards the tumor site.

Overall, these results demonstrate that T cells expressing the chemokine receptor CX3CR1 show increased homing towards tumors producing the specific ligand, and highlight the critical issue of a gradient between peripheral tissues and the tumor.

## Discussion

Adoptive T cell transfer is increasingly used as anti-cancer therapy but its efficacy is still to be optimized. Several strategies have been pursued and introduced to enhance the success of adoptive T cell immunotherapy. Whereas most current approaches to genetically modify tumor-specific T cells are aimed at enhancing T cell persistence, tumor recognition or activation of innate immunity [38, 39], our study focused on the approach to increase the homing of T cell to the tumor site.

There is strong evidence that chemokine ligands expressed in the tumor micro-environment contribute to the local recruitment of leukocytes [40–42]. The production of specific chemokines has been related to the presence of particular leukocyte subsets. For instance, CCL5 and CXCL9 and CXCL10 contributed to the recruitment of CD8 T cells in a mouse model of spontaneous melanoma [43]. Hong and colleagues have shown that the chemotherapeutic drugs dacarbazine, temozolomide, and cisplatin enhanced the expression of some chemokines in melanoma patients, which in turn correlated with improved immune control of tumors [44]. Vice versa, a decreased expression of chemoattractants is associated with reduced infiltration of CD8 T cells and tumor relapse following T cell therapy [45]. Notably, in a subset of patients with melanoma metastases, lack of chemoattractants coincides with limited migration of CD8 T cells [43].

The introduction of chemokine receptors in genetically engineered T cells has the potential to enhance the homing of adoptively transferred effector cells (T cells or NK cells) leading to significantly improved tumor-targeting [19]. Some studies have reported the transduction of chemokine receptors such as CXCR2, CXCR3, CCR2b or photo-activatable CXCR4 [20, 22–24]. When gene-engineered to express CXCR2, T cells displayed enhanced trafficking towards tumor cells secreting the corresponding chemokine CXCL1 [18]. Also, in xenograft tumor models of mesothelioma and neuroblastoma, the genetic introduction of CCR2 in T cells resulted in increased T cell infiltration in tumors secreting CCL2 and was associated with significantly increased anti-tumor activity [21].

We chose to transduce the CX3CR1 receptor (among many chemokine receptors) as several tumor types produce the unique ligand Fractalkine and, of importance, this chemokine ligand is expressed as a membrane-bound molecule, serving as an adhesion receptor [35, 36]. Furthermore, Fractalkine is produced by activated endothelial cells [46, 47], which may be of advantage for the recruitment of adoptively transferred T cells from the blood circulation to the tumor site. The CX3CR1 receptor is mainly present in tissue resident macrophages (e.g. in the intestinal mucosa), in a subset of NK cells, at low levels in T cells, and preferentially expressed in activated Th1 lymphocytes [34]. Of note, we recently found in human colorectal tumors, that T lymphocytes at the tumor front, as well as within the tumor, do not express the CX3CR1 receptor and therefore cannot be recruited at tumor site, in spite of ligand expression by cancer cells [31].

In the present study, transduction of CX3CR1 into CD3/IL-2 activated T cells was achieved quite efficiently and resulted in an appropriately located surface receptor that was functionally active in migration assays in vitro. The adoptive transfer of CX3CR1-transduced T cells in tumor-bearing mice indeed resulted in improved homing towards Fractalkine-expressing tumors. This was confirmed by mRNA, flow cytometry of human TIL in mouse tumors, and by immunohistochemistry.

As a source of ligand-expressing tumor cells, we have selected the human colorectal cell line NCI-H630 that constitutively produces low levels of Fractalkine. In parallel experiments we used also another tumor cell line (RKO) transduced with Fractalkine and producing very high levels of the chemokine, also as a shed soluble ligand. Interestingly, under these conditions, the homing of CX3CR1-T cells was not enhanced, compared to eGFP-T cells. Mice bearing Fractalkine-transduced RKO tumors had very high serum levels of circulating ligand, which likely have interfered with the correct recruitment of adoptively transferred T cells, while serum levels of NCI-H630-formed tumors were negligible, indicating an exclusively local expression. These findings are consistent with the concept that localized expression of the chemo-attractant, and hence a proper blood-tissue gradient, is a crucial pre-requisite for the effective recruitment of adoptively transferred effectors from circulation to tumors.

In our study, we used polyclonal T cells generated by CD3/IL-2 activation. Of note, we did observe slower tumor growth in mice injected with CX3CR1-T cells; this finding is of considerable importance because it indicates that even T cell effectors with unknown tumor specificity could be partially effective, if sufficient numbers reach the tumor.

The chemokine-transduction approach could be easily applied to tumor-specific T cells generated by genetic modification with specific T cell receptors, hopefully improving their therapeutic efficacy [48]. This approach may provide an important new avenue in “personalized cancer therapy” taking advantage of defined chemokine signatures within the tumor microenvironment and may further benefit cancer patients undergoing ACT. Furthermore, a targeted localized delivery of specific cytotoxic effector T cells may have the potential to prevent common adverse effects associated to adoptive T cell immunotherapy, such as cytokine storm, and represents a novel method to improve T cell cytotoxicity, infiltration and ultimately anti-tumor efficacy.

## Conclusions

Overall, our results demonstrate that T lymphocytes engineered with the chemokine receptor CX3CR1 have enhanced homing towards CX3CL1-producing tumors, when adoptively transferred in vivo in mice. Increased T cell trafficking and infiltration in tumor tissues resulted in decreased tumor growth. Our results also established the importance of a correct blood-tumor chemokine gradient for the successful recruitment of adoptively transferred T cells.

## Methods

### Patients and tissue specimens

Patients included in this study underwent resective surgery for colorectal cancer at the Clinical and Research Institute Humanitas (Milan, Italy) from 1997 to 2003. The study was approved by the Institute Ethical Committee, and written informed consent was obtained from patients.

### Animal experiment and adoptive transfer

6–8 weeks old female NSG (NOD/SCID/IL-2rg^−/−^) mice (Jackson Laboratories) were used. Procedures involving animals and their care confirmed to institutional guidelines in compliance with national (4D.L. N.116, G.U., suppl. 40, 18-2-1992) and international law and policies (EEC Council Directive 2010/63/EU, OJ L 276/33, 22.09.2010; NIH Guide for the Care and Use of Laboratory Animals, U.S. National Research Council, 2011). Mice (6–7 mice for each group) received subcutaneous injection into the flanks with 3 × 10^6^ of viable cells (NCI-H630). For adoptive transfer experiments, 3 × 10^6^ of transduced T lymphocytes were transferred in each mouse through tail vein injection after 18 days of tumor growth and for thrice at three days of interval. At last mice were sacrificed and tumors were harvested. The experiment was designed based on our previous study [49] and pilot experiments.

### Cells and reagents

HCT116, NCI-H630 and RKO cells were cultured in RPMI 1640 medium supplemented with 10 % FBS, 2 mM Ultraglutamine and 100U/ml penicillin/streptomycin (Lonza, BioWhittaker). The packaging cell line Phoenix-A and HEK 293 T cells were cultured in DMEM medium (Lonza, BioWhittaker) supplemented with 10 % FBS, 2 mM Ultraglutamine and 100U/ml penicillin/streptomycin. TNF-α and IFN-γ (Peprotech) were used for in vitro stimulation of cell lines.

Monoclonal antibodies used in this study were as follows: anti-CD3 mAB OKT3 (EB16-0037-85, eBioscience), PE-conjugated anti-human CX3CR1 (D070-5, MBL), allophycocyanin (APC) conjugated anti-human CD3 (555342, BD Biosciences), APC-Cy7 conjugated mouse anti-human CD8 (557852, BD Biosciences), PerCP-Cy5.5-conjugated anti-human CD3 (560835, BD Biosciences), APC conjugated anti-human CD45 (560973, BD Biosciences), PE-Cy7 conjugated anti-human CD4 (557852, BD Biosciences). Other reagents included RetroNectin (T100-A, Takara); recombinant human IL-2 (202-IL, R&D Biosystems); Collagenase Type I (C0130, Sigma); DNAse I (D5025, Sigma), Hyaluronidase (H3506, Sigma).

### Cloning Fractalkine/Fractalkine receptor

To generate retroviral vector for fractalkine receptor, *CX3CR1-eGFP* was amplified by PCR using human glioblastoma tumor sample as template (based on our previous study [50]) and cloned between the restriction sites *EcoRI* and *NotI* into pMP71 retroviral vector (kind gift from Dr. Wolfgang Uckert, MDC Berlin Germany). Fractalkine gene was cloned and overexpressed in RKO cell line using lentiviral mediated transduction as described earlier [31].

### Lymphocyte culture, activation, transduction and expansion

PBMC from healthy donors were isolated by centrifugation through Ficoll-Isopaque (density = 1.077 g/cm3; Amersham Pharmacia Biotech, Uppsala, Sweden). Human T lymphocytes of healthy donors were activated with anti-CD3 mAb and transduced with retrovirus harboring either eGFP (Mock) or CX3CR1-eGFP transgenes. Moloney murine leukemia retroviruses were produced by cocultures of 293 T and Phoenix-A cells. Cells were calcium phosphate transfected with transgenes, the helper vectors pHIT60 MLV GAG/POL, and pCOLT-GALV ENV. The transduction protocol of human T cells was optimized and described previously. Transduced primary human T cells were cultured in RPMI 1640 medium supplemented with 25 mM HEPES, 200 mM L-glutamine, 10 % human serum, antibiotics, and 360 IU/mL human rIL-2 (Proleukin; Chiron, Amsterdam, The Netherlands) and stimulated every 2 weeks with a mixture of irradiated allogeneic feeder cells as described elsewhere [51, 52].

### Flow cytometry

T Lymphocytes were washed and incubated in ice cold washing buffer (phosphate-buffered saline (PBS) containing 2 % FCS) with anti-CD3, anti-CD4, anti-CD45, anti-CD8, and anti-CX3CR1-PE for 30 min. Cells were then washed and fixed with 2 % PFA to acquire and analyzed with FACSCanto or LSR-II (BD Biosciences) and FlowJo (Treestar) software.

### ELISA

Equal number of cells (2 × 10^6^) cells were seeded in a monolayer fashion in a six well plate and allowed to incubate in CO_2_ incubator at 37 °C. Supernatant was collected post 24 h of incubation. ELISA for CX3CL1/Fractalkine was performed using ELISA Kit (R&D) according to manufacturer instructions. Tumor lysate for ELISA was collected after mincing small tumor fragments followed by incubation in lysis buffer and extraction of supernatant.

### RT-PCR and Quantitative Real Time qPCR

Total RNA was isolated either from cells or tumors using TRI reagent (Ambion) and quantified with nanodrop. DNAse treatment (Turbo DNA-free kit, Ambion) was performed to avoid genomic DNA contamination. 1 μg of total RNA was reverse-transcribed using the High-Capacity cDNA Archive kit (Applied Biosystems). cDNA was analyzed by SYBER Green based Quantitative Real-Time PCR on ABI Prism® 7900HT Fast Real Time PCR System (Applied Biosystem). 18S was used as internal control to normalize. All gene specific primers were domestically designed. The sequences of the primer pairs are as follows:

**Table:** 

18S	(F-CGCCGCTAGAGGTGAAATTC; R-CTTTCGCTCTGGTCCGTCTT)
CD3	(F-GAGTTCGCCAGTCGAGAGCT; R-TCATCTTCTCGATCCTTGAGGG)
CD4	(F-CTTCTTCTGTGTCAGGTGCCG; R-AGGAGTCTCTTGATCTGAGACATCC)
CD8	(F-CTGAGCAACTCCATCATGTACTTCA; R-GTCGTGGTGGGCTTCGC)
CX3CR1	(F-GGGACTGTGTTCCTGTCCAT; R-GACACTCTTGGGCTTCTTGC)
CX3CL1	(F-TCTGCCATCTGACTGTCCTG; R-TGATGTTGCATTTCGTCACA)

### T cell migration

Lymphocyte migration assay was performed using 24-well plate in triplicate by using 5.0 μm pore size positron emission tomography (PET) membrane (BD Biosciences, Bedford, MA). The upper chamber was loaded with transduced T lymphocytes and the lower bottom chamber was filled with culture medium supplemented with different concentration of rhCX3CL1 (365-FR-025, R&D Systems). After 18 h of incubation, the migrated cells to the lower side of the inserts were harvested and counted.

### Immunofluorescence

NCI-H630 cell line were cultured on coverslips coated with Poly-L-Lysine, washed in PBS and fixed in 4 % PFA for 15’. After 2 washes in 2 % BSA in PBS, cells were incubated with the primary antibody rabbit anti-human CX3CL1 (Torrey Pines Biolabs), diluted in 2 % BSA, 0.1 % TritonX-100, 0.1 % glycine, 5 % Normal Goat Serum in PBS. After 3 washes in washing buffer (0.2 % BSA, 0.05 % Tween 20 in PBS), cells were incubated with secondary antibody Alexa goat anti-rabbit 488 (Invitrogen) for 1 h at room temperature. After 4 washes in washing buffer, DAPI was used to stain nuclei. Coverslip was mounted with Fluor Preserve^TM^ Reagent (Calbiochem) and the images were acquired with a laser scanning confocal microscope (FluoView FV1000; Olympus). For image analysis, Imaris X64 7.0.2 Software (Bitplane, AG) was used.

### Tumor digestion and isolation of TIL

Triple enzyme method was used to digest the tumor. Briefly, harvested tumors were minced with scalpels into very small fragments and mixed with 5 ml of HBSS containing Collagenase (10 mg/ml), Hyaluronidase (1 mg/ml) and DNase (200 U/ml) and incubated at 37° on stir plate for 1–2 h. Tissue was further dissociated by passing through 100 μm nylon mesh filter unit into falcon tubes to pellet and wash in fresh HBSS. Tumor infiltrating lymphocytes (TIL) were isolated based on their density and collected at the interface between 40 % and 80 % discontinuous Percoll gradient.

### Immunohistochemistry

Formalin-fixed, paraffin-embedded tissues were deparaffinised and endogenous peroxidase was blocked with 3 % hydrogen peroxide for 20 min at room temperature. For CD3 staining, tumor sections were exposed to an antigen retrieval procedure with 1 mM EDTA buffer. Reactive sites were identified by exposure to Anti-Rat Polymer HRP kit (Biocare Medical, CA USA) for 30 min at room temperature. Immunoperoxidase staining was then performed by using diaminobenzidine as a chromogen (DAB+chromogenX-50, ChemMate, DakoCytomation, Carpinteria, CA, USA). The slides were finally counterstained with haematoxylin (Harris Hematoxilyn, DiaPath, Microstain Division, Martinengo, Bergamo, Italy). For quantitative evaluation, a computer-aided image analysis software was used, able to discriminate the immunostained area on the basis of RGB color segmentation, and to calculate the percentage of immunoreactive area as a fraction of the total area digitally captured. For Fractalkine/CX3CL1 immunostaining, paraffin-blocks of human tumors with a diagnosis of colorectal cancer were obtained from the Humanitas Pathology archive and stained as detailed in our previous study [31].

### Statistical analysis

Prism software (v6.0a; Graphpad) was used to conduct appropriate statistical procedures, as noted in the individual figure legends. P value <0.05 was considered significant unless noted otherwise.

## Additional files

Additional file 1: Figure S1. Constitutive and stimulated expression of Fractalkine/CX3CL1 by human colorectal cancer cell lines. (A) Fractalkine/CX3CL1 mRNA expression in different colorectal cancer cell lines cultured with or without IFN-γ (1000U/ml) and TNF-α (50 ng/ml) for 24 h. (B) Fractalkine/CX3CL1 expression in RKO cells transduced with the cDNA of CX3CL1 (RKO-CX3CL1) or mock-transduced RKO-Mock. (C) Tumor growth of RKO-Mock and RKO-CX3CL1 cells in NSG mice. (JPG 1379 kb) Additional file 2: Figure S2. Transduction of human T lymphocytes with eGFP/CX3CR1-eGFP gene. (A) Population of CD3+ and of CD4+/CD8+ T cells post retroviral transduction of eGFP/CX3CR1 or -eGFP gene after 3 days of in vitro culture with hIL-2 medium. (JPG 1522 kb) Additional file 3: Figure S3. Lymphocyte infiltration in the lungs of tumor-bearing mice after adoptive transfer. Flow cytometry analysis of T lymphocytes entrapped in the lungs of mice receiving adoptive transfer of CX3CR1-T cells or GFP-T cells (3 mice per group). (A) Proportion of CD3+ T lymphocytes among CD45+ cells in the disaggregated lungs from mice bearing RKO-Mock or RKO-CX3CL1 tumors. (B) Proportion of CD3+ T lymphocytes among CD45+ cells in the disaggregated lungs from mice bearing NCI-H630 tumors. (JPG 1661 kb)

## Abbreviations

ACT: adoptive T cell therapy
CARs: chimeric antigen receptors
eGFP: enhanced Green Fluorescent Protein
TCRs: T cell receptors
TIL: tumor-infiltrating lymphocytes
